# Total Flavonoids of *Rhizoma Drynariae* Mitigates Aflatoxin B1-Induced Liver Toxicity in Chickens via Microbiota-Gut-Liver Axis Interaction Mechanisms

**DOI:** 10.3390/antiox12040819

**Published:** 2023-03-28

**Authors:** Shucheng Huang, Luxi Lin, Shiqiong Wang, Wenli Ding, Chaodong Zhang, Aftab Shaukat, Bowen Xu, Ke Yue, Cai Zhang, Fang Liu

**Affiliations:** 1 College of Veterinary Medicine, Henan Agricultural University, Zhengzhou 450046, China; huang.sc@henau.edu.cn (S.H.); linmo0726@163.com (L.L.);; 2 College of Food Science and Technology, Henan Agricultural University, Zhengzhou 450002, China; 3 National Center for International Research on Animal Genetics, Breeding and Reproduction (NCIRAGBR), Huazhong Agricultural University, Wuhan 430070, China; 4 Laboratory of Environment and Livestock Products, Henan University of Science and Technology, Luoyang 471023, China

**Keywords:** aflatoxin B1, antioxidation, bile acid, Chinese medicinal herb, ferroptosis, gut microbiota

## Abstract

Aflatoxin B1 (AFB1) is a common mycotoxin that widely occurs in feed and has severe hepatotoxic effects both in humans and animals. Total flavonoids of *Rhizoma Drynaria* (TFRD), a traditional Chinese medicinal herb, have multiple biological activities and potential hepatoprotective activity. This study investigated the protective effects and potential mechanisms of TFRD against AFB1-induced liver injury. The results revealed that supplementation with TFRD markedly lessened broiler intestinal permeability by increasing the expression of intestinal tight junction proteins, as well as correcting the changes in gut microbiota and liver damage induced by AFB1. Metabolomics analysis revealed that the alterations in plasma metabolites, especially taurolithocholic acid, were significantly improved by TFRD treatment in AFB1-exposed chickens. In addition, these metabolites were closely associated with [*Ruminococcus*], ACC, and GPX1, indicating that AFB1 may cause liver injury by inducing bile acid metabolism involving the microbiota–gut–liver axis. We further found that TFRD treatment markedly suppressed oxidative stress and hepatic lipid deposition, increased plasma glutathione (GSH) concentrations, and reversed hepatic ferroptosis gene expression. Collectively, these findings indicate that ferroptosis might contribute to the hepatotoxicity of AFB1-exposed chickens through the microbiota–gut–liver axis interaction mechanisms; furthermore, TFRD was confirmed as an herbal extract that could potentially antagonize mycotoxins detrimental effects.

## 1. Introduction

Aflatoxin B1 (AFB1) is the largest proportion of known aflatoxins, with the highest degree of mycotoxin contamination [1]. The International Agency for Research on Cancer (IARC) evaluated AFB1 as one of the most toxic chemical carcinogens known to disrupt the normal function of multiple organs and tissues [2,3]. It has been reported that poultry might be most susceptible to AFB1 toxicity [4]. AFB1 exposure significantly impaired poultry production performance and reproductive performance, such as meat quality, feed intake, body weight, and egg production, which caused great economic losses to the poultry breeding industry [4,5]. Recently, AFB1 was reported to cause liver oxidative stress, inflammation, ferroptosis, and eventually liver cancer [6,7]. Thus, it is important to find some green harmless substances that can prevent or treat AFB1 poisoning.

The gastrointestinal tract is the first place that encounters mycotoxins, as the absorption rate of aflatoxin is approximately 90% in poultry [8]. The intestinal mucosal barrier, a semipermeable structure, consists of biological, physical, chemical, and immune barriers that jointly prevent the intestinal wall from being invaded by toxicants and bacteria. Among them, biological and physical barriers both play an essential role in the development and function of the intestinal barrier to ensure intestinal health [9]. Once the intestinal barrier is disrupted, many toxins enter the bloodstream and are distributed to their target organs, producing toxic damage [10]. Several findings have revealed that AFB1 can impair gastrointestinal mucosal barrier function and disrupt the gut microbiota [11,12]. The balance of the gut microbiota is vital for animal digestion and absorption, immune regulation, etc. [13]. Moreover, disturbance in the microbiota can lead to an increase in harmful microorganisms and the adverse metabolic product lipopolysaccharide (LPS); furthermore, this disturbance can exacerbate the toxic damage of mycotoxins to host health [14,15]. Therefore, maintaining gut microbiota homeostasis may be key for ameliorating AFB1 toxicity damage.

The liver, as an important metabolic organ, is also a major target organ affected by aflatoxin toxicity. For example, AFB1 can induce inflammatory damage, oxidative stress, apoptosis, and autophagy to cause liver function damage [1,16,17]. Furthermore, a recent study found through transcriptome sequencing analysis that liver damage caused by AFB1 exposure may be connected to the expression pathways of PPAR (peroxisome proliferator-activated receptor) and ferroptosis [18]. Ferroptosis is a term that describes a unique modality of regulated cell death, which is provoked by compromised redox and antioxidant machinery and disseminated by lipid peroxidation reactions. Moreover, its initiation and execution are regulated through several cellular metabolic pathways, including cellular redox homeostasis, by modulating the cellular antioxidant mechanisms, mitochondrial glutamine metabolism, lipid metabolism, and glucose metabolism [19,20,21]. AFB1 induces oxidative stress to produce reactive oxygen species (ROS) and malondialdehyde (MDA), which can induce lipid peroxidation and may play a key role in liver ferroptosis [1,22]. The primary mechanism of ferroptosis is the catalysis of lipid peroxidation on the cell membranes, which causes extremely high expression of polyunsaturated fatty acids (PUFAs) under the action of divalent iron or ester oxygenase and suppresses the antioxidant system (mainly glutathione (GSH, an important antioxidant defense agent)) and selenoprotein glutathione peroxidase 4 (GPX4, an important antioxidant enzyme), resulting in cell death [23,24,25]. Moreover, PPARα is involved in regulating the expression of many important genes related to lipid and lipoprotein metabolism [26]. However, the role of gut microbiota disturbance combined with the regulatory mechanism of ferroptosis in AFB1-triggered liver injury in broiler chickens remains unclear.

Since the immense damage to the chicken industry caused by AFB1, many research studies have been carried out on the physical and chemical removal or degradation of mycotoxins, and excellent results have been achieved. In recent years, there have been many studies on the improvement of mycotoxin toxicity by Chinese herbal medicine, among which flavonoid extracts play an important role [27,28]. In our previous study, we explored the protective and antioxidant effects of the total flavonoids of *Rhizoma Drynariae* (TFRD), obtained from the dried root of *Rhizoma Drynariae*, on liver damage caused by AFB1 [1]. Therefore, in this study, we systematically explored the protective effects and potential mechanisms of TFRD against AFB1-induced liver injury in terms of the gut microbiota, intestinal barrier, and ferroptosis and provided a theoretical basis for the development of TFRD as a nutraceutical, adjuvant drug, and feed additive.

## 2. Materials and Methods

### 2.1. Chemicals

AFB1 (#2A1A08) was sourced from Pribolab Biological Engineering Co., Ltd. (Qingdao, China). TFRD (#K20798) had been acquired from Xi’an Kailai Biological Engineering Co., Ltd. (Xi’an, China). The compositional analysis of TFRD is shown in Figure S1 and Table S1, and its main component was detected by liquid chromatography-mass spectrometry (LC-MS) analysis as *Rutin* (97.82%) [29]. Hematoxylin and eosin (HE) and Periodic Acid-Schiff (PAS) were purchased from Servicebio^®^ (Wuhan, China). The 4% paraformaldehyde was purchased from Yantai Shuangshuang Chemical Co., Ltd. (Yantai, China). TRIzol solution was acquired from Takarabio Technology Co., Ltd. (Beijing, China; #AKF0726A).

### 2.2. Animal Care and Experimental Design

Ninety male Arbor Acres broiler chickens (one day old) were purchased from Kaifeng Xingda Poultry Co., Ltd. (Kaifeng, China). Animals were allocated in cages with a controlled environment, and feed and water were provided ad libitum. After 3 weeks of acclimatization, the broiler chickens were randomly assigned to 3 groups (*n* = 30 each, Figure S2A) as follows: the control group, the AFB1 group, and the AFB1 + TFRD (A + T) group. Each treatment was replicated 3 times with 10 broiler chickens per replicate. Among them, the CON group was administered 4% ethanol solution by injection into the ingluvies, and AFB1 (100 μg/kg body weight, dissolved in 4% ethanol solution) was injected into the ingluvies of broiler chickens in the AFB1 and A + T groups for 7 days to establish the AFB1 exposure model. Since AFB1 is highly toxic, we ensured ingluvies injection could accurately handle the dose, and, at the same time, it can well prevent the toxin from spreading into the environment. Additionally, the ingluvies is a unique organ of avian animals, which is directly under the skin and is more convenient for drug injection than the gastrointestinal tract. Furthermore, the broiler chickens in the A + T group received a basal diet supplemented with TFRD at a dosage of 125 mg/kg. The concentration and dosage of AFB1 and TFRD and the use of solvents were referred to by Lin et al. [1] and Cao et al. [11]. The Animal Welfare and Research Ethics Committee of the College of Veterinary Medicine, Henan Agricultural University approved and supported all experiments (Permit No: HNND2022030818).

### 2.3. Sample Collection

Following 7 days of treatment, the broiler chickens fasted for 12 h, and blood was collected into anticoagulant tubes and divided into 3 layers after centrifugation (3000× *g*/15 min, 4 °C). The most upper plasma was stored at −80 °C for experimental analysis. Then, broilers were sacrificed by cervical dislocation, 5 mm mid-duodenum section and 5 × 5 mm liver section were fixed in 4% paraformaldehyde for subsequent histopathological observation. The remaining liver tissue and duodenal mucosa were individually snap-frozen in liquid nitrogen and then stored at −80 °C without thawing for the following molecular experiments. Additionally, duodenum contents were stored at −80 °C for gut microbiota sequencing analysis.

### 2.4. Histological Analysis

HE staining slides were prepared from 4% paraformaldehyde-fixed and paraffin-embedded liver and duodenum. Typical pathologic changes in the liver and duodenum were investigated under a microscope (Motic^®^ BA600-4, Xiamen, China). Villus length and crypt depth were measured on slides where the duodenum was well oriented, using ImageJ software (#version 1.53e) developed by the National Institutes of Health (NIH) (Bethesda, Rockville, MD, USA).

PAS staining was used to locate duodenal goblet cells to assess the changes in intestinal metabolism and response to diseases. Paraffin sections were dewaxed with xylene, stained with PAS solution, and dehydrated with anhydrous ethanol. ImageJ software was used to quantify goblet cells.

### 2.5. Oil Red O Staining for Detection of Liver Lipid Accumulation

A qualitative measure of lipids in the liver was obtained by staining with Oil Red O (ORO; Zhengzhou Biopple Biological Technology Co., Ltd., Zhengzhou, China). Stained liver tissue sections were examined at 10 × magnification under a Nikon Eclipse E100 microscope. ImageJ software was used to quantify ORO lipid staining of the basal zone.

### 2.6. Biochemical Analysis

Six plasma samples were randomly taken from each group for the determination of the activities of liver-function-related enzymes, including alanine aminotransferase (ALT, #C009-2-1) and aspartate aminotransferase (AST, #C010-2-1); the activities of antioxidant related enzymes, including glutathione peroxidase 1 (GPX-1, #A005-1-2), superoxide dismutase (SOD, #A001-1-2), and catalase (CAT, #A007-1-1); the levels of reduced glutathione (GSH, #A006-2-1) and oxidized glutathione (GSSG, #A061-1-1); the concentration of malondialdehyde (MDA, A003-1-2); and the levels of lipid-metabolism-related indicators, including total cholesterol (TC, #A111-1-1), high-density lipoprotein cholesterol (HDL-C, #A112-1-1), triglyceride (TG, #A110-1-1), and low-density lipoprotein cholesterol (LDL-C, #A113-1-1). All detection kits were purchased from Nanjing Jiancheng Bioengineering Institute (Nanjing, China), and the procedure was performed as referenced in our previous studies [1,30]. In addition, plasma diamine oxidase (DAO) activity was detected using an ELISA kit (#CK-E69214; Shanghai Mlbio Biotechnology Co., Ltd., Shanghai, China).

### 2.7. Determination of Related Gene Expression Levels

Real-time PCR (RT-qPCR) analysis was conducted as formerly described [28]. Briefly, the total quantity of RNA was isolated from duodenal mucosa and liver tissue using TRIzol reagent, and cDNA was prepared using a kit from Vazyme, Nanjing, China. Subsequently, SYBR Green I PCR Master Mix carried out the procedure on an RT-qPCR instrument (Analytik Jena, qTOWER, Jena, Germany) using specific primers (Table S2). The 2^−△△CT^ method was used to analyze data and normalize to glyceraldehyde-3-phosphate dehydrogenase (GAPDH) expression.

### 2.8. Western Blot Analysis

Protein concentrations in the duodenal mucosa lysate were determined by bicinchoninic acid (BCA) assay. Proteins were loaded onto an SDS-PAGE gel (#WLA013a, Wanleibio Co., Ltd., Shenyang, China) and electrophoresed and analyzed via Western blotting using antibodies against Occludin (1:1000; #WL01996), Claudin-1 (1:1500; #WL03073), and β-actin (1:1000; #WL01372), followed by HRP goat anti-rabbit IgG secondary antibody (1:5000; #WLA023a, Wanleibio, Shenyang, China). The bands were detected with chemiluminescence kits (#CW0049s, CWBIO, Beijing, China). An Image Station 440CF was used to record chemiluminescence. ImageJ software was used to analyze the results. 

### 2.9. DNA Extraction, Sequencing of the 16S rRNA Gene and Microbiota Data Analysis

Microbial genomic DNA was extracted from duodenal content samples using the FastDNA SPIN kit (#116540600) according to the kit’s protocol and then stored at −20 °C before further analysis (MP Biomedicals, Santa Ana, CA, USA). Raw data were analyzed using the QIIME2 platform, as previously described [31]. Additionally, α and β diversities were determined in QIIME2. Principal coordinates analysis (PCoA) plots were produced using the “ape” packages of the particular R software (v3.2.0). Differentially abundant genera among groups were recognized using linear discriminant analysis (LDA) and effect size (LEfSe) analysis. Only bacterial taxa attaining the LDA threshold of 3.5 along with average relative abundances greater than 0.01% were analyzed. The 16S rRNA gene sequence datasets involved in this study have been hosted in the National Center for Biotechnology Information Sequence Read Repository (accession no. PRJNA917784).

### 2.10. LC-MS/MS Analyses

The plasma samples were thawed and vortexed for 10 s for LC-MS/MS analysis. Concisely, 50 μL of each sample was added to 300 μL of 20% acetonitrile methanol as the internal standard. The samples were centrifuged at 4 °C at 12,000 r/min for 10 min and then allowed to stand at −20 °C for 30 min. The samples were centrifuged again for 3 min, and the supernatant was taken into the inner liner of the corresponding sample bottles for on-machine analysis. The obtained data were processed using Analyst 1.6.3 software (Wuhan, China).

### 2.11. Statistical Analysis 

All data were analyzed employing IBM SPSS Statistics (R26.0.0.0, Inc., Chicago, IL, USA) for variance examination and Student’s *t*-test; GraphPad Prism (Version 8.0.2, Graphpad, La Jolla, CA, USA) was used to construct column or line charts. One-way ANOVA was used to evaluate the significant differences among the three groups. Almost all experimental data are presented as the mean ± standard error of the mean (S.E.M.), and *p* < 0.05 corresponds to statistical significance.

## 3. Results

### 3.1. TFRD Reverses the Alteration of Gut Microbiota Caused by AFB1 Exposure

**Figure 1:**
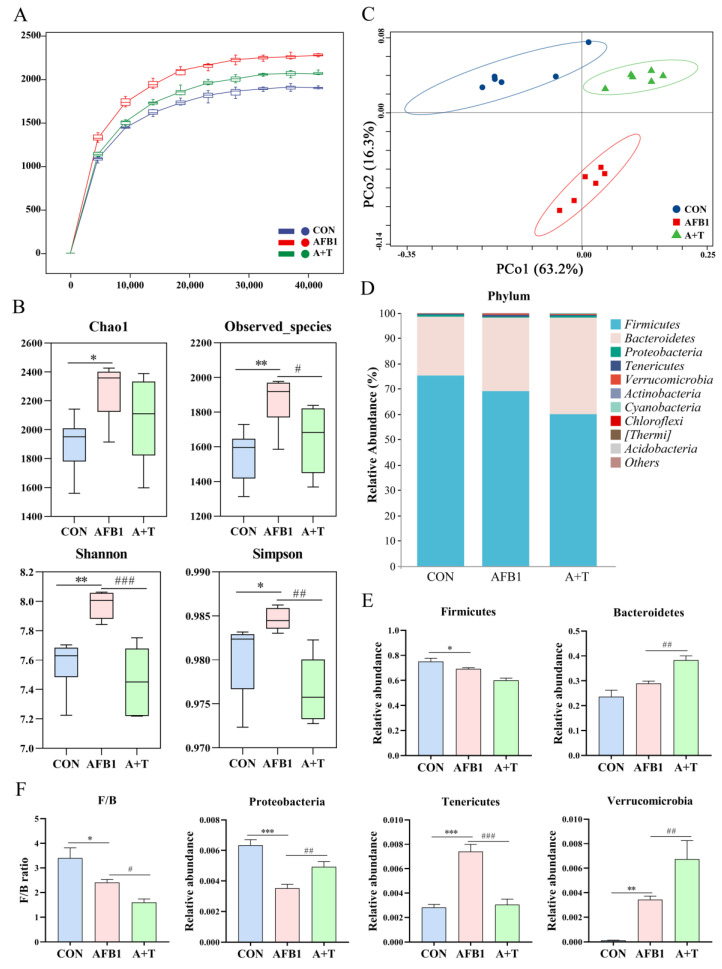
The effect of TFRD on AFB1-induced abnormal variations in the composition of the gut microbiota. (**A**) Rarefaction curve. (**B**) Alpha diversity analysis of gut microbiota in experimental broiler chickens. (**C**) PCoA based on the Weighted–UniFrac distance matrix of OTUs. (**D**) Relative abundance of gut microbial composition at the phylum level (top 10). (**E**,**F**) The effects of AFB1 and TFRD on the relative abundance of microbiota at the phylum level. F/B, *Firmicutes*/*Bacteroidetes* ratio. Differences between groups were analyzed by One-way ANOVA. The presented values are the mean ± SEM (*n* = 6). Differences were considered significant at (*) *p* < 0.05, (**) *p* < 0.01, and (***) *p* < 0.001 compared to the CON group and at (#) *p* < 0.05, (##) *p* < 0.01, and (###) *p* < 0.001 compared to the AFB1 group.

The animal encounters AFB1 in feed mainly through ingestion, and the microbiota in the gastrointestinal tract is the first to be exposed. AFB1 absorption is principally in the small intestine, while the duodenum is the intestinal segment with the most efficient absorption [32]. Next, the effect of AFB1 on the gut microbiota was assessed by collecting the contents of the duodenum of broiler chickens after AFB1 exposure for 16S rRNA sequencing. As illustrated in Figure 1A, the species rarefaction curve tended to flatten, indicating that the sequencing quantity and depth conformed to the requirements of subsequent analysis. Moreover, the alpha diversity indexes Chao1 (*p* < 0.05), Shannon (*p* < 0.01), Observed_species (*p* < 0.01), and Simpson (*p* < 0.05) were dramatically elevated in the AFB1 group compared to that in the CON group (Figure 1B), indicating that AFB1 exposure changed the abundance and diversity of the gut microbiota. TFRD treatment markedly reduced the Observed_species (*p* < 0.05), Shannon (*p* < 0.001), and Simpson indexes (*p* < 0.01) compared to those in the AFB1 group (Figure 1B). The β-diversity of the gut microbiota, as assessed by PCoA analysis, showed that the duodenal microbiota of the AFB1 group was appreciably distinct from the other two groups, whereas the A + T group was close to the CON group (Figure 1C). 

**Figure 2:**
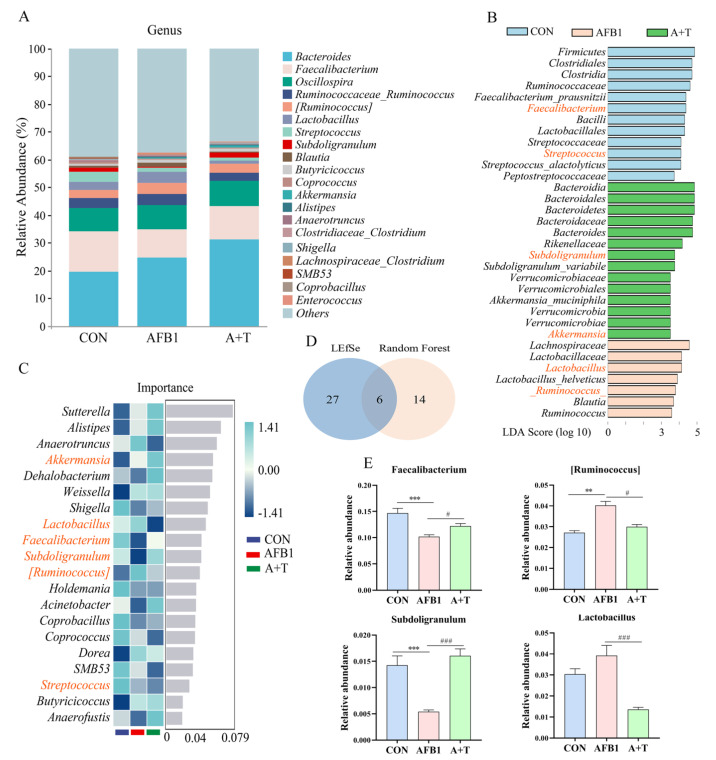
TFRD protects against AFB1–induced abnormal changes in the gut microbiota at the genus level. (**A**) Relative abundance of gut microbial composition at the genus level (top 20). (**B**) The differential genera screened by LEfSe analysis and random forest analysis were analyzed by Venn diagram, and the coincidence part was the significantly different genera. Blue indicates CON group, red indicates AFB1 group, and green indicates A + T group. (**C**) Random forest analysis (top 20 in importance). Blue indicates CON group, red indicates AFB1 group, and green indicates A + T group. (**D**) Venn diagram analysis of differential genera. (**E**) Four genera changed significantly in the overlap of the Venn diagram. Differences between groups were analyzed by One–way ANOVA. The presented values are the mean ± SEM (*n* = 6). Differences were considered significant at (**) *p* < 0.01, and (***) *p* < 0.001 compared to the CON group and at (#) *p* < 0.05, and (###) *p* < 0.001 compared to the AFB1 group.

The gut microbiota composition was further analyzed at both the phylum and genus levels. The composition of the broiler gut microbiota community at the phylum level (top 10) and genus level (top 20) was evidently different among the different treatment groups (Figure 1D and Figure 2A). At the phylum level, the highest abundance was found in the phyla *Firmicutes* and *Bacteroidetes*. After AFB1 exposure, there was a significant decrease in the abundance of *Firmicutes* and *Proteobacteria* (*p* < 0.05 and *p* < 0.001, respectively) and the *Firmicutes*/*Bacteroidetes* (F/B) ratio (*p* < 0.05) and a significant increase in the abundance of *Tenericutes* (*p* < 0.001) and *Verrucomicrobia* (*p* < 0.01) compared to that in the CON group broilers (Figure 1E,F). Compared with the AFB1 group, there was a significantly elevated relative abundance of *Bacteroidetes*, *Proteobacteria*, and *Verrucomicrobia* (all *p* < 0.01), a markedly reduced relative abundance of *Tenericutes*, and a reduced F/B ratio in the A + T group broilers (*p* < 0.001 and *p* < 0.05, respectively; Figure 1F). 

At the genus level, LEfSe analysis was performed on the gut microbiota with a current LDA threshold of 3.5, histograms were used to identify the species that were significantly enriched in each group, and a total of 33 genera were screened (Figure 2B). Among them, *Lachnospiraceae, Lactobacillaceae, Lactobacillus, Lactobacillus_helveticus, [Ruminococcus], Blautia*, and *Ruminococcus* were drastically increased in the AFB1 groups. The cladogram showed a significant increase in the relative abundance of the phylum *Firmicutes* in the CON group compared with that in the other groups (Figure S2B). Subsequently, the microbiota from the most important genera (top 20) were screened using random forest analysis (Figure 2C). Furthermore, the genera with LDA values exceeding 3.5 and the top 20 most important genera are presented in a Venn diagram (Figure 2D). Compared to the CON group, we observed that AFB1 mainly disrupted the abundance of bacteria associated with bile acid metabolism, such as *Faecalibacterium*, *Subdoligranulum*, *Lactobacillus*, and [*Ruminococcus*]. AFB1 exposure notably decreased the relative abundance of *Faecalibacterium* and *Subdoligranulum* (all *p* < 0.001) but apparently increased the relative abundance of [*Ruminococcus*] (*p* < 0.01) compared with the CON group (Figure 2E). Interestingly, it was noticed that the relative abundance of *Faecalibacterium* and *Subdoligranulum* was markedly elevated (*p* < 0.05 and *p* < 0.001, respectively), and *Lactobacillus* and [*Ruminococcus*] were significantly lower (*p* < 0.001 and *p* < 0.05, respectively) in the A + T group in comparison to the AFB1 group, showing that TFRD supplementation inhibited AFB1-induced changes in gut microbiota composition. These findings suggested that TFRD supplementation significantly reversed the dysbiosis of the gut microbiota induced by AFB1 exposure in broiler chickens.

### 3.2. TFRD Improves the Intestinal Barrier Dysfunction Caused by AFB1 Exposure

**Figure 3:**
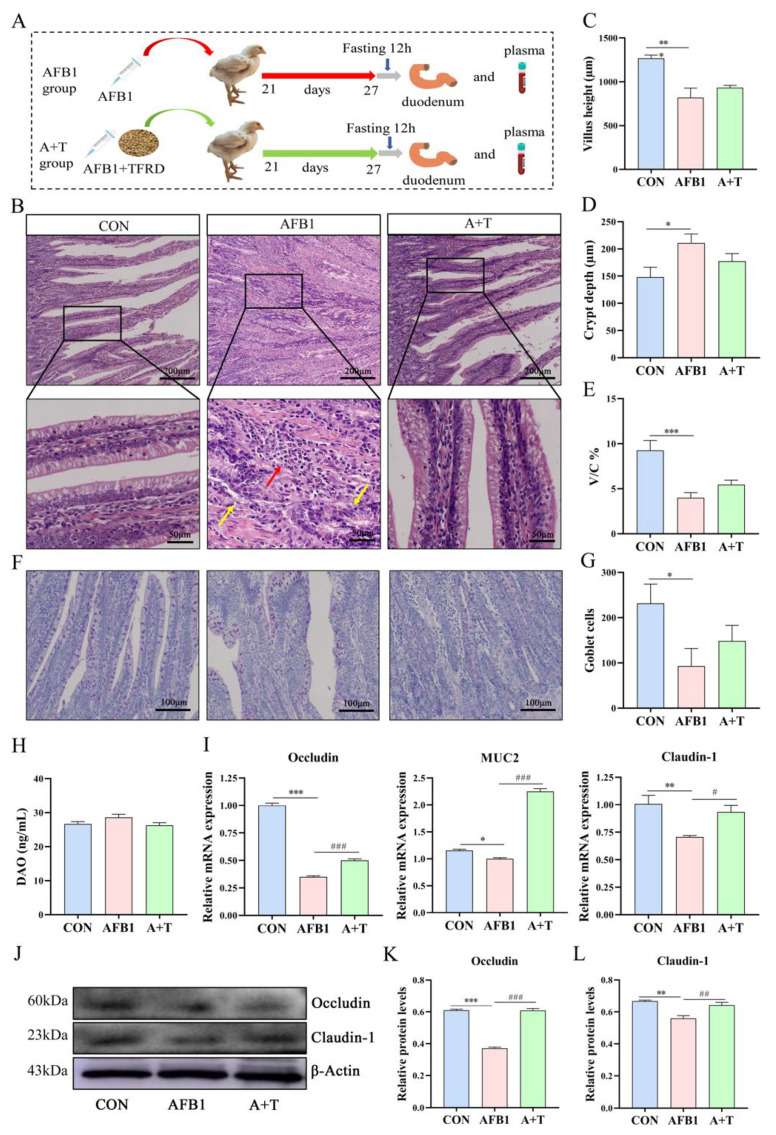
TFRD improved AFB1-induced intestinal mucosal barrier injury. (**A**) Broiler chicken ingluvies injected with AFB1 or fed with TFRD interfered with intestinal mucosal barrier function. (**B**) Hematoxylin and eosin (HE) staining results of the duodenum. Scale bar = 200 μm for × 4 magnification; scale bar = 50 μm for × 20 magnification (yellow arrows: intestinal villi rupture; red arrow: inflammatory cells). (**C**–**E**) Villus height, crypt depth, and the ratio of villus height/crypt depth (V/C) of the duodenum. (**F**) Periodic Acid-Schiff (PAS) staining results of the duodenum (scale bar = 100 μm for × 10 magnification). (**G**) Goblet cell of the duodenum. (**H**) The activity of plasma diamine oxidase (DAO). (**I**) mRNA expression levels of *occludin*, *MUC2*, and *claudin-1*. (**J**–**L**) Occludin and Claudin-1 protein expression levels. Differences between groups were analyzed by One–way ANOVA. The presented values are the mean ± SEM (*n* = 4). Differences were considered significant at (*) *p* < 0.05, (**) *p* < 0.01, and (***) *p* < 0.001 compared to the CON group, at (#) *p* < 0.05, (##) *p* < 0.01 and (###) *p* < 0.001 compared to the AFB1 group.

Morphological observations of intestinal tissue and tight junction-related protein expression assays were performed to evaluate the properties of TFRD on AFB1–induced intestinal barrier dysfunction in broilers (Figure 3A). The histology of HE and PAS stain was shown in Figure 3B,F. The duodenal villi were structurally intact, and the goblet cells were evenly distributed in the crypts in the CON group. However, disorder or even rupture of the intestinal villus and considerable infiltration of inflammatory cells at the break were observed in the AFB1 group (Figure 3B), with a significant decrease in villus height (*p* < 0.001), villus height/crypt depth (V/C) ratio (*p* < 0.001), and goblet cells (*p* < 0.05) and a significant increase in crypt depth (*p* < 0.05; Figure 3C–E,G). Compared with the AFB1 group, the A + T group alleviated the above negative changes, and the goblet cell maturation rate was increased by 59%. In addition, the plasma DAO level, an indicator of the integrity and damage of the intestinal mechanical barrier, did not change considerably among the three groups (Figure 3H). 

Furthermore, the results of tight junction-related protein expression showed that TFRD supplementation dramatically reversed the AFB1-induced reduction of *occludin*, *MUC2*, and *claudin-1* mRNA expression levels in duodenal mucosa (*p* < 0.001, *p* < 0.001, and *p* < 0.05, respectively; Figure 3I). Compared with the CON group, the AFB1 group exhibited markedly decreased protein levels of Occludin (*p* < 0.001) and Claudin-1 (*p* < 0.01, Figure 3J–L). Moreover, the tight junction protein level was elevated in the A + T group in comparison to the AFB1-exposed group. Together, these results revealed that AFB1 exposure impairs intestinal mucosal barrier function and that TFRD can alleviate these adverse effects.

### 3.3. TFRD Regulates Plasma Metabolism

**Figure 4:**
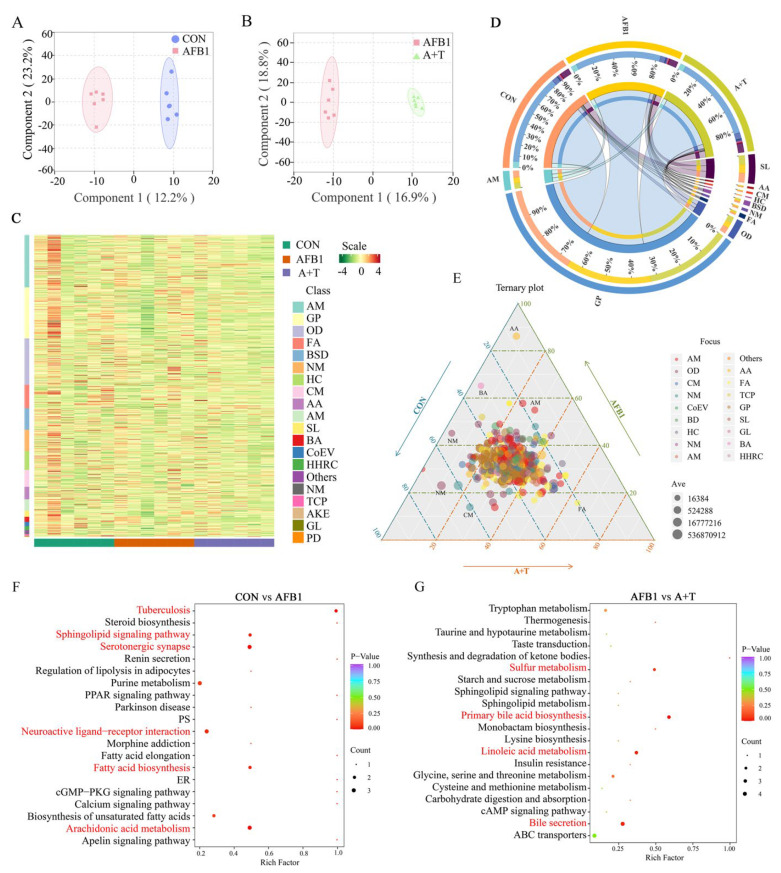
TFRD protects against AFB1–induced alterations in plasma metabolite. (**A**) OPLS–DA of the CON and AFB1 groups. (**B**) OPLS–DA of the AFB1 and A + T groups. (**C**) Heatmap of metabolite content clustering. (**D**) Metabolite abundance analysis by LC–MS/MS. (**E**) Ternary analysis shows the relative abundance of metabolites in different groups. AM, Amino acid and its metabolomics; GP, Glycerol phospholipids; OD, Organic acid and their derivatives; FA, Fatty acyl; NM, Nucleotide and its metabolomics; BSD, Benzene and substituted derivatives; HC, Heterocyclic compounds; CM, Carbohydrates and its metabolites; AA, Alcohol and amines; SL, Sphingolipids; BA, Bile acids; CoEV, CoEnzyme and vitamins; HHRC, Hormones and hormone-related compounds; TCP, Tryptamines, Cholines, Pigments; AKE, Aldehyde, Ketones, Esters; and PD, Pteridines and derivatives. (**F**,**G**) Metabolic pathway analysis based on significantly differential metabolites from the CON, AFB1, and A + T groups. The color of the dots is *p* value, and the redder the color, the more significant the enrichment. Differences between groups were analyzed by Student’s *t*-test. PS, Parathyroid hormone synthesis, secretion and action; ER, Endocrine and other factor-regulated calcium reabsorption.

Previous studies have reported that gut microbiota disorders can lead to dysregulation of bile acid metabolism, and it is well known that bile acids are important substances in the interconnection between the liver and intestine [33,34]. We next performed a metabolomic analysis of broiler plasma samples by LC–MS/MS to assess the effect of AFB1 absorption through the gastrointestinal tract into the circulatory system on blood metabolites. A total of 773 metabolites were identified in the CON group, AFB1 group, and A + T group. Orthogonal partial least squares discriminant analysis (OPLS-DA, variable importance in the project (VIP ≥ 1)) was used to screen the plasma metabolites with significant differences among the CON, AFB1, and A + T groups (Figure 4A, B). Subsequently, all metabolites were clustered, mainly as amino acid, glycerol phospholipids (GP), organic acid, fatty acyl (FA), benzene, and their derivatives. (Figure 4C). Among them, the most abundant metabolite group was GP, followed by sphingolipids (SLs), as shown in the Circos plot (Figure 4D). Moreover, the Ternary plot further showed the dominant metabolites in the different groups. Partial metabolites were discretely distributed in each group, illustrating the differential metabolites that emerged among the three groups (Figure 4E). 

KEGG pathway enrichment was performed based on the differential metabolite results, and KEGG enrichment maps of differential metabolites demonstrated the overall changes in metabolic pathways. As shown in Figure 4F, the pathways significantly enriched in the CON and AFB1 groups were tuberculosis, serotonergic synapse, arachidonic acid metabolism, sphingolipid signaling pathway, fatty acid biosynthesis, and neuroactive ligand–receptor interaction (*p* < 0.05). The pathways enriched in the AFB1 and A + T groups were primary bile acid biosynthesis, linoleic acid metabolism, bile secretion, and sulfur metabolism (*p* < 0.05, Figure 4G).

**Figure 5:**
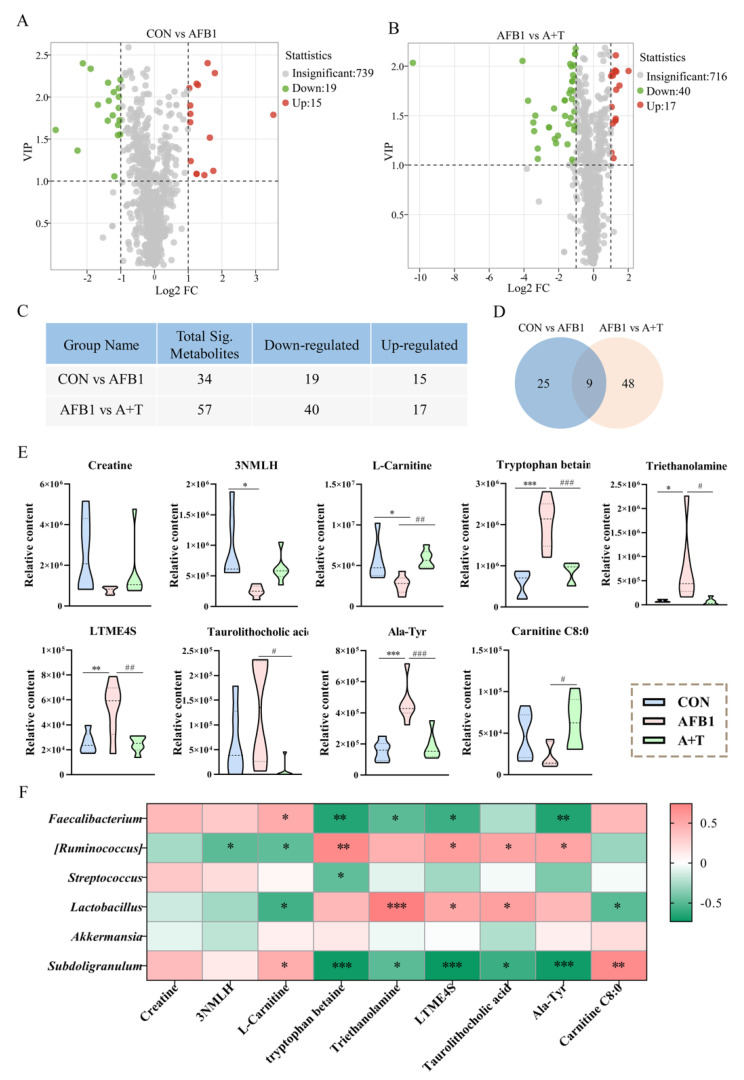
The differential plasma metabolites after AFB1 exposure were correlated with the gut microbiota. (**A**,**B**) Volcano map of differential metabolites between the CON, AFB1, and A + T groups. Difference screening condition: FC ≥ 2 and FC ≤ 0.5, VIP ≥ 1. Differences between groups were analyzed by Student’s *t*-test. (**C**) Statistical table of differential metabolites. (**D**) Venn diagram analysis of differential metabolites in each group (FC ≥ 2 and FC ≤ 0.5, VIP ≥ 1). (**E**) The relative contents of core differential metabolites in the 3 groups (*n* = 6). Differences between groups were analyzed by One-way ANOVA. The presented values are the mean ± SEM. Differences were considered significant at (*) *p* < 0.05, (**) *p* < 0.01, and (***) *p* < 0.001 compared to the CON group and at (#) *p* < 0.05, (##) *p* < 0.01, and (###) *p* < 0.001 compared to the AFB1 group. (**F**) Association analysis of differential bacteria with the marker differential metabolites by Pearson correlation. The asterisks (* *p* < 0.05, ** *p* < 0.01 and *** *p* < 0.001) indicate statistically significant correlations. 3NMLH, 3–N–Methyl–L–Histidine; LTME4S, L–tyrosine methyl ester 4–sulfate.

The volcano plot presented the changes in differential metabolites among the three groups (Figure 5A,B). Among them, the number of identified differential metabolites in the CON and AFB1 groups and the AFB1 and A + T groups were 34 (including 15 metabolites upregulated and 19 metabolites downregulated) and 57 (including 17 metabolites upregulated and 40 metabolites downregulated), respectively (Figure 5C). Moreover, Venn diagram analysis was used to identify a total of 9 core differential metabolites among the 3 groups (Figure 5D), 4 of which were lower and 5 of which were higher in the AFB1 group than in the CON group. For example, 3–N-methyl–L–histidine and L–carnitine were significantly reduced (*p* < 0.05), while tryptophan betaine, triethanolamine, L-tyrosine methyl ester 4–sulfate, and alanine–tyrosine (Ala–Tyr) were elevated (*p* < 0.001, *p* < 0.05, *p* < 0.01, and *p* < 0.001, respectively) in the AFB1 group compared with the CON group (Figure 5E). In the A + T group, the abundances of L-carnitine and carnitine C8:0 were elevated compared to the AFB1 group (*p* < 0.01 and *p* < 0.05, respectively), whereas the abundances of tryptophan betaine, triethanolamine, L–tyrosine methyl ester 4–sulfate, taurolithocholic acid, and Ala–Tyr in the plasma were markedly lower than those in the AFB1 group (*p* < 0.001, *p* < 0.05, *p* < 0.01, *p* < 0.05, and *p* < 0.001, respectively). 

Pearson correlation analysis was performed to explore potential correlations between the microbiota and metabolites. The results showed that *Subdoligranulum* was significantly positively correlated with L–carnitine (r = 0.477, *p* < 0.05) and carnitine C8:0 (r = 0.666, *p* < 0.01; Figure 5F) and significantly negatively correlated with taurolithocholic acid (r = −0.581, *p* < 0.05). However, *Lactobacillus* was markedly negatively correlated with L-carnitine (r = −0.562, *p* < 0.05) and carnitine C8:0 (r = −0.492, *p* < 0.05) and markedly positively correlated with taurolithocholic acid (r = 0.550, *p* < 0.05). [*Ruminococcus*] also exhibited a significant negative correlation with L-carnitine (r = −0.479, *p* < 0.05) and a significant positive correlation with taurolithocholic acid (r = 0.526, *p* < 0.05). In addition, a positive correlation was identified between *Faecalibacterium* and L-carnitine (r = 0.486, *p* < 0.05). These results indicated that AFB1 exposure induced dysregulation of plasma metabolites closely related to the gut microbiota.

### 3.4. TFRD Alleviates AFB1-Induced Liver Injury

**Figure 6:**
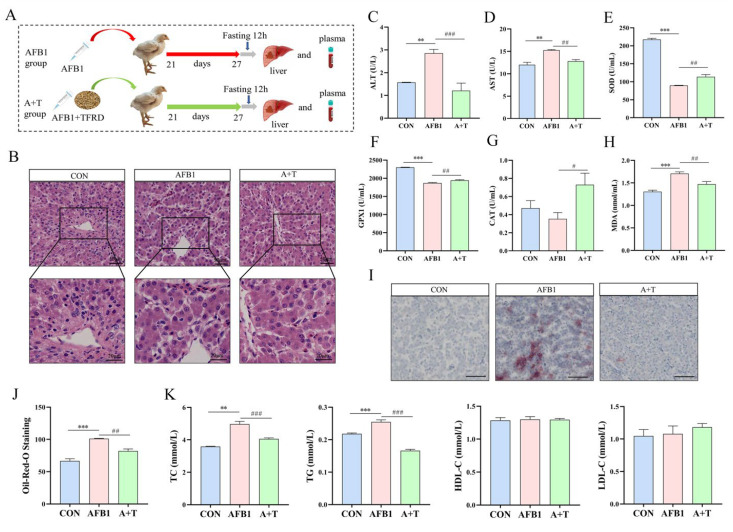
TFRD improves AFB1-induced liver injury. (**A**) To evaluate liver performance, broilers were treated with an ingluvies injection of AFB1 or feed supplemented with TFRD. (**B**) Hematoxylin and eosin (HE) staining of liver tissue. Scale bar = 50 μm for 20× magnification; scale bar = 20 μm for 40× magnification. (**C**,**D**) Plasma alanine aminotransferase (ALT) and aspartate aminotransferase (AST) activity. (**E**–**H**) Plasma antioxidant enzyme superoxide dismutase (SOD), glutathione peroxidase 1 (GPX1), catalase (CAT) activities, and oxidation product malondialdehyde (MDA) levels. (**I**) Oil Red O (ORO) staining of liver tissue (scale bar = 100 μm for ×10 magnification). (**J**) The lipid droplet number of the liver with oil red O staining. (**K**) The levels of plasma total cholesterol (TC), triglyceride (TG), high-density lipoprotein cholesterol (HDL-C), and low-density lipoprotein cholesterol (LDL-C). Differences between groups were analyzed by One-way ANOVA. The presented values are the mean ± SEM (*n* = 6), Differences were considered significant at (**) *p* < 0.01 and (***) *p* < 0.001 compared to the CON group and at (#) *p* < 0.05, (##) *p* < 0.01, and (###) *p* < 0.001 compared to the AFB1 group.

As shown in Figure 6B, HE staining of liver tissue showed that the hepatocytes in the CON group were closely and regularly arranged. In contrast, the hepatocytes in the AFB1 group displayed granular degeneration, swollen hepatocytes, ruptured nuclear membranes, and even nuclear disappearance. Supplementation of TFRD in AFB1-exposed broilers gradually compacted the hepatocytes. Next, plasma ALT and AST enzyme activities were higher in the AFB1 group than in the CON group (all *p* < 0.01). In contrast, both enzyme activities were markedly decreased in the A + T group (*p* < 0.001 and *p* < 0.01, respectively; Figure 6C,D). Furthermore, the plasma MDA level was elevated, while the activities of the antioxidant enzymes SOD and GPX1 were reduced (all *p* < 0.001), indicating that oxidative damage occurred in broiler chickens after AFB1 exposure (Figure 6E–H). Additionally, supplementation with TFRD relieved AFB1-induced oxidative damage in broiler chickens, according to decreased MDA levels and increased SOD, GPX1, and CAT activities (*p* < 0.01, *p* < 0.01, and *p* < 0.05, respectively).

The findings of ORO staining of liver tissue showed that AFB1 exposure resulted in a significant elevate in lipid droplets in hepatocytes in comparison to the CON group (*p* < 0.001, Figure 6I). The amount of lipid droplet deposition was appreciably higher in the AFB1 group than in the A + T group (*p* < 0.01, Figure 6J). Likewise, we detected the blood lipid levels and found that the plasma TC and TG concentrations in the AFB1 group were markedly increased in comparison to those in the CON group (*p* < 0.01 and *p* < 0.001, respectively; Figure 6K), and the concentrations of both in the A + T group were reduced prominently compared with those in the AFB1 group (all *p* < 0.001). In addition, HDL-C and LDL-C levels did not change significantly between groups. These results indicated that AFB1 exposure caused liver tissue damage, hepatocyte lipid droplet aggregation, and even steatosis, which might promote abnormal lipid metabolism in the liver that could be alleviated by dietary supplementation with TFRD.

### 3.5. TFRD Protects against AFB1-Induced Hepatic Ferroptosis and Dyslipidaemia

**Figure 7:**
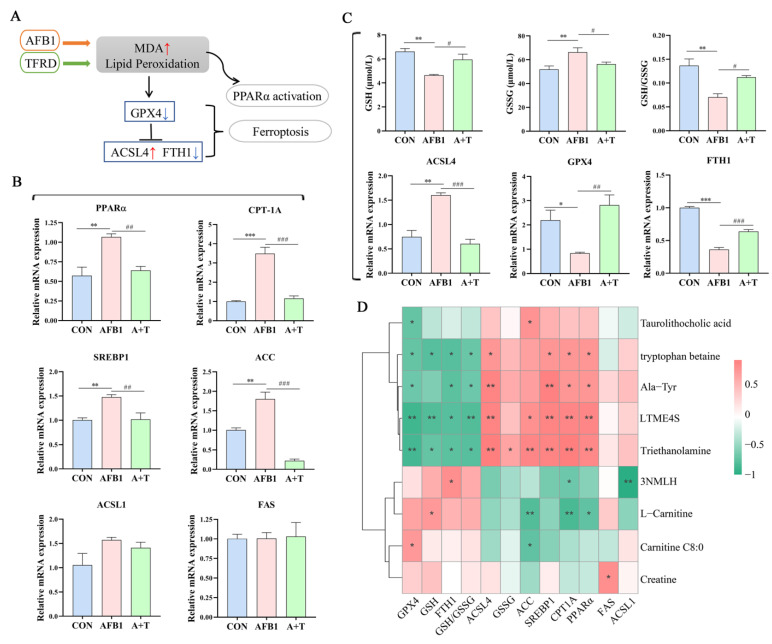
TFRD ameliorates AFB1-induced hepatic ferroptosis and abnormal lipid metabolism. (**A**) Lipid peroxidation promotes ferroptosis and PPARα activation. (**B**) The mRNA expression levels of key lipid metabolism genes (*PPARα*, *CPT-1A*, *SREBP1*, *ACC*, *FAS*, and *ACSL1*) in liver. (**C**) Plasma GSH and GSSG levels, the GSH/GSSG ratio, and the mRNA expression levels of key ferroptosis genes (*ACSL4*, *GPX4*, and *FTH1*) in liver. GSH, glutathione; GSSG, oxidized glutathione. Differences between groups were analyzed by One-way ANOVA. The presented values are the mean ± SEM (*n* = 6). Differences were considered significant at (*) *p* < 0.05, (**) *p* < 0.01, and (***) *p* < 0.001 compared to the CON group and at (#) *p* < 0.05, (##) *p* < 0.01, and (###) *p* < 0.001 compared to the AFB1 group. (**D**) Association analysis of key ferroptosis parameters and lipid metabolism genes with marker differential metabolites by Pearson correlation. The asterisks (* *p* < 0.05, and ** *p* < 0.01) indicate statistically significant correlations. 3NMLH, 3-N-Methyl-L-Histidine; LTME4S, L-tyrosine methyl ester 4-sulfate.

Oxidative damage is a prominent feature of AFB1-induced liver injury, and ferroptosis is closely related to oxidative stress, while lipid peroxidation is the key to ferroptosis (Figure 7A). PPARα mainly regulates the expression of lipid metabolism-related genes. In this study, a significant increase was found in the mRNA expression of PPARα in the AFB1 group compared with the CON group (*p* < 0.01, Figure 7B), while its expression level was markedly decreased in the A + T group (*p* < 0.01). Furthermore, the expression levels of the CPT-1A, SREBP1, and ACC genes were all considerably elevated in the AFB1 group in comparison to the CON group (*p* < 0.001, *p* < 0.01, and *p* < 0.01, respectively), and the ACSL1 and FAS genes showed no significant difference in the CON, AFB1, and A + T groups. As expected, the expression levels of the CPT-1A, SREBP1, and ACC genes were decreased in the A + T group compared to the AFB1 group (*p* < 0.001, *p* < 0.01, and *p* < 0.001, respectively). It was apparent that AFB1 exposure led to abnormal hepatic lipid metabolism, which can aggravate oxidative damage in chickens.

According to the published literature, GSH is an important antioxidant in the body, and reducing the level of GSH can promote ferroptosis [35]. As shown in Figure 7C, AFB1 exposure significantly reduced the plasma GSH concentration and the GSH/GSSG ratio (all *p* < 0.01), while TFRD noticeably reversed the upregulation of the plasma GSSG concentration caused by AFB1 exposure (*p* < 0.05). To further investigate the occurrence of ferroptosis, we examined the mRNA expression of key regulatory genes for ferroptosis in the liver, such as ACSL4, a biomarker of ferroptosis, and GPX4, a central inhibitor. The results demonstrated that AFB1 exposure markedly elevated the mRNA expression levels of *ACSL4* (*p* < 0.01) and reduced that of *GPX4* (*p* < 0.05) and *FTH1* (*p* < 0.001). By comparison, supplementation with TFRD significantly reversed the above alteration manifested by increased gene expression levels of *GPX4* (*p* < 0.01) and *FTH1* (*p* < 0.001) and decreased gene expression levels of *ACSL4* (*p* < 0.001). These findings indicated that decreased antioxidant capacity and abnormal hepatic lipid metabolism may promote ferroptosis.

Moreover, Pearson correlation analysis revealed that the key factors of ferroptosis and different metabolites in plasma were mainly negatively correlated (Figure 7D). The GSH level and the expression of *GPX4* and *FTH1* were significantly positively correlated with carnitine C8:0 (r = 0.695, *p* < 0.05), L-carnitine (r = 0.703, *p* < 0.05), and 3-N-methyl-L-histidine (r = 0.770, *p* < 0.05). There was a significant positive correlation between *ACSL4* and tryptophan betaine, Ala-Tyr, L-tyrosine methyl ester 4-sulfate, and triethanolamine (r = 0.720, 0.851, 0.847, and 0.889; *p* < 0.05, *p* < 0.01, *p* < 0.01, and *p* < 0.01; respectively). Interestingly, most of the lipid metabolism genes were significantly positively correlated with differentially expressed plasma metabolites. For example, the gene expression levels of *PPARα*, *CPT-1A*, *SREBP1*, and *ACC* were positively correlated with triethanolamine (r = 0.815, 0.908, 0.842, and 0.826, respectively; all *p* < 0.01). In contrast, L-carnitine was negatively correlated with *ACC*, *CPT-1A*, and *PPARα* (r = −0.805, −0.843, and −0.743; *p* < 0.01, *p* < 0.01, and *p* < 0.05; respectively). Additionally, taurolithocholic acid was negatively correlated with *GPX4* (r = −0.716, *p* < 0.05) and positively correlated with *ACC* (r = 0.731, *p* < 0.05). It is evident that alterations in plasma metabolites under AFB1 exposure are closely related to the regulatory mechanisms of hepatic ferroptosis and abnormal lipid metabolism.

## 4. Discussion

**Figure 8:**
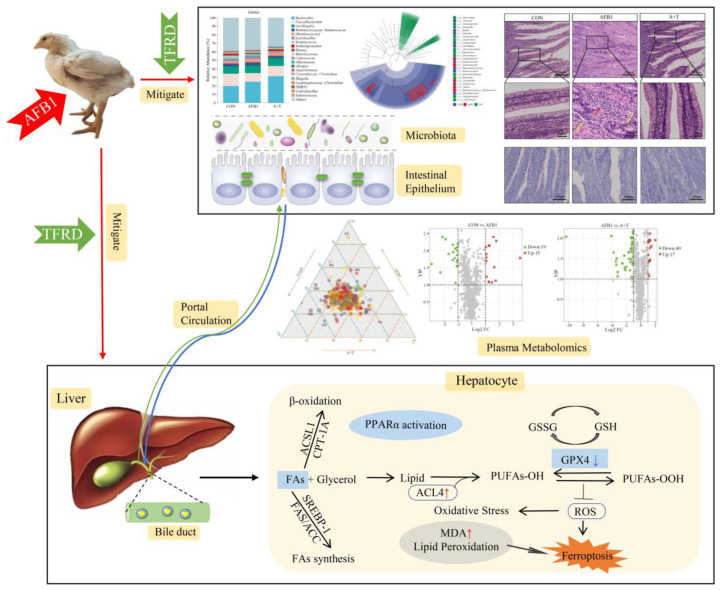
TFRD mitigates AFB1–induced liver toxicity in chickens via microbiota–gut–liver axis interaction mechanisms.

AFB1 is a class of highly toxic mycotoxins, and the waste of feed ingredients and decreased animal performance caused by AFB1 contamination have brought heavy economic losses to the livestock industry [36]. As a flavonoid herbal medicine extract, TFRD has various bioactive effects, including immunomodulatory, anti-inflammatory, antioxidant, and anti-apoptotic effects [1,5,29,31]. In this present experiment, the main active ingredient of TFRD is *Rutin*. It has been reported that *Rutin*, as an antioxidant, can reduce DNA damage caused by AFB1 [37]. In recent years, the metabolic pathways involved in mitigating AFB1-induced liver injury by TFRD have remained elusive, despite numerous studies on the molecular mechanisms of AFB1 toxicity focusing on liver injury [6,38]. Our findings demonstrated that the beneficial effects of dietary supplementation with TFRD mitigate the AFB1-induced liver injury via modulation of the gut microbiota and plasma metabolite balance, repair of the intestinal barrier, and suppression of ferroptosis (Figure 8). This is the main benefit of the potent antioxidant properties of TFRD.

The gastrointestinal tract is the primary source of digestion and absorption of nutrients in the animal body [39]. Moreover, the gastrointestinal tract is one of the critical immune organs of the body, which also helps resist the invasion of exogenous pathogenic factors and maintains the homeostasis of the intestinal environment [40]. It is normal for the gut microbiota, the host, and the external environment to establish a dynamic equilibrium, and the type and quantity of the gut microbiota are relatively stable [41]. However, AFB1 exposure disrupts the gut microbiota [42]. *Lactobacillus* can inhibit the reproduction of spoilage and pathogenic bacteria in the gut and reduce blood ammonia and cholesterol content [43,44]. An excessive amount of *Lactobacillus* can also cause an imbalance in the gut microbiota, which affects normal intestinal peristalsis. Our study found a trend of increased intestinal *Lactobacillus* abundance following AFB1 exposure. Interestingly, plasma TC content also showed an increase with AFB1 interference, indicating that gut microbiota might affect plasma metabolites by altering blood circulation following AFB1 interference.

The intestinal physical barrier is a complete intestinal epithelial structure composed of intestinal epithelial cells and tight junctions [45]. The tight junction is a complex protein system formed by the interaction of a series of transmembrane proteins and peripheral proteins, which can be regulated in many ways [46]. Occludin and Claudin-1 are the major constituent proteins of tight junctions, and their protein and gene expression levels are suppressed after exposure to mycotoxins [15,47,48]. Gut villus height, crypt depth, and the V/C ratio are vital indicators for evaluating animal gut development and functional status [49]. Our results showed that AFB1 ingestion decreased the V/C ratio, indicating reduced digestion and absorption, and increased the chances of diarrhea and slow growth in broilers, which is consistent with the findings of Gao et al. [12]. Additionally, the protective effect of TFRD on gut barrier function is the restoration of intestinal tissue morphology and the promotion of the expression of tight junctions.

Moreover, many reports have shown that the gut microbiota and bile acids interact, and bile acid receptors expressed in the intestine, such as farnesoid X receptor, vitamin D3 receptor, and Takeda G protein receptor 5, are involved in the regulation of intestinal barrier function [50]. Normally, bile acids are stored in the gallbladder in combination with glycine and taurine and secreted into the intestinal lumen when animals eat [51,52]. The majority of bile acids (approximately 95%) are reabsorbed in the small intestine (ileum) and are secreted and restored to the liver through the portal vein, while the unabsorbed bile acids are processed by the gut microbiota to form secondary bile acids [53,54]. Our study found plasma bile acid overload in AFB1-exposed broilers, while bile acid overload caused oxidative damage to hepatocytes [55]. Once the homeostasis of gut microbiota, an important regulator of bile acid metabolism, is disrupted, bile acid metabolism is also affected. At the same time, disrupted bile acid metabolism can lead to an inflammatory response in the intestinal epithelium and even increase intestinal permeability [56]. However, how gut microbiota imbalance and intestinal barrier function inhibition are involved in liver injury remains unknown. Through plasma metabolomic analysis, we found that AFB1 exposure mainly induced upregulation of fatty acid biosynthesis, while TFRD promoted downregulation of primary bile acid biosynthesis and bile secretion. AFB1 exposure increased plasma and liver lipid content [57]. In this present study, AFB1 ingestion increased hepatic lipid droplets as well as plasma TG and TC contents, as demonstrated by Chen et al. [58]. Likewise, the level of the secondary bile acid taurolithocholic acid in plasma was markedly reduced under TFRD treatment. These findings may serve as a new pathway for alleviating liver injury caused by AFB1 exposure via the microbiota–gut–liver axis.

Ferroptosis has been recognized as a lipid peroxidation-mediated non-apoptotic form of cell death [59]. An increasing number of studies have indicated the involvement of ferroptosis in the pathogenesis of liver diseases, such as non-alcoholic steatohepatitis (NASH) [60,61]. Hence, targeting ferroptosis may provide a new beneficial strategy for treating liver disease. The GSH pathway plays a vital role in antioxidant defense. The metabolic protein GPX4 can convert GSH into GSSG and reduce lipid peroxides (PL-OOH) to corresponding alcohols (PL-OH), thereby controlling lipid peroxidation and protecting cells from ferroptosis [62,63]. In the present study, increased MDA and depletion of GSH and GPX4 after AFB1 exposure were essential markers of ferroptosis [60]. Thus, ferroptosis is closely related to oxidative stress and the inhibition of ferroptosis by TFRD may occur through the antioxidant system [35]. Furthermore, the KEGG enrichment analysis of the plasma metabolites showed that arachidonic acid metabolism was significantly enriched in the AFB1 group. As a PUFA, arachidonic acid is vulnerable to peroxidation and promotes ferroptosis under the induction of AFB1 [64].

PPARα regulates the body’s glucose and lipid metabolism balance and is mainly expressed in the liver [65]. After PPARα takes effect in the liver, it can regulate fatty acid transport and β-oxidation as well as lipid metabolism [66]. PPARα functions as a crucial regulatory receptor in the nucleus that is activated by endogenous and exogenous factors. It controls the expression of downstream signaling pathways that produce fatty acids, including the fatty acid synthesis genes SREBP-1, FAS, and ACC and the fatty acid oxidation genes ACSL1 and CPT-1A [66,67]. Stimulation of PPARα induces an increase in the β-oxidation rate, and fatty acids are oxidized to acetyl-CoA under the action of ACSL1, and then acetyl-CoA is catalyzed to generate acylcarnitine by CPT-1A. The protein encoded by ACSL1 is only one isoenzyme of the long-chain fatty acid-CoA ligase family, in which all isozymes have the function of converting free long-chain fatty acids to fatty acyl-CoA esters, thus exerting pivotal roles in lipid biosynthesis and fatty acid degradation [68,69]. SREBP1 is a critical transcription factor that is involved in controlling lipid metabolism in cells. It primarily regulates the expression of essential enzyme genes within cholesterol biosynthesis, fatty acid synthesis, and lipid regeneration [70]. Importantly, SREBP1 and its regulated fatty acid synthesis pathway are expressed and active at low levels in normal tissues and cells. In contrast, they are enormously elevated in tissues with abnormal lipid metabolism [71]. Therefore, this may be the reason why the expression level of the SREBP1 gene increased significantly after AFB1 exposure. TFRD supplementation results indicated that TFRD could reduce the abnormal expression of key factors in fatty acid oxidation or synthesis that were induced by AFB1 exposure.

## 5. Conclusions

In short, this work showed that TFRD has a protective effect against AFB1-induced liver injury and imbalances in microbiota–gut–liver axis homeostasis through an integrated interaction, including regulation of the gut microbiota and intestinal barrier function, correction of lipid as well as bile acid metabolism, and inhibition of hepatic oxidative stress and ferroptosis. These findings provide evidence that the microbiota–gut–liver axis is involved in AFB1-induced liver injury by causing intestinal barrier damage and hepatic ferroptosis. Future research should further investigate the links between the gut microbiota and hepatic ferroptosis in AFB1-induced liver injury.

## Data Availability

The data are contained within the article and supplementary materials.

## Supplementary Materials

The following supporting information can be downloaded at: https://www.mdpi.com/article/10.3390/antiox12040819/s1, Figure S1: Composition and percentage of components of TFRD; Table S1: Composition and percentage of components of TFRD; Figure S2: (A) Scheme of experiments. (B) Cladogram generated from LEfSe analysis of gut microbiota (LDA score > 3.5).
