# Supplementary material for: Total Flavonoids of Rhizoma Drynariae Mitigates Aflatoxin B1-Induced Liver Toxicity in Chickens via Microbiota-Gut-Liver Axis Interaction Mechanisms
Source: Antioxidants (Basel). 2023 Mar 28;12(4):819. doi: 10.3390/antiox12040819 (PMC10134996; doi:10.3390/antiox12040819)
Supplement: Supplementary file 1 [file antioxidants-12-00819-s001.zip › antioxidants-2246698-supplementary.pdf]

Total flavonoids of *Rhizoma Drynariae* mitigate aflatoxin B1-induced liver toxicity in chickens via microbiota-gut-liver axis interaction mechanisms

Shucheng Huang[1], Luxi Lin[1], Shiqiong Wang[2], Wenli Ding[1], Chaodong Zhang[1], Aftab Shaukat[3], Bowen Xu[1], Ke Yue[1], Cai Zhang[4], Fang Liu[1*]

[1] College of Veterinary Medicine, Henan Agricultural University, Zhengzhou 450046, China;

[2] College of Food Science and Technology, Henan Agricultural University, Zhengzhou 450002, China;

[3] National Center for International Research on Animal Genetics, Breeding and Reproduction (NCIRAGBR), Huazhong Agricultural University, Wuhan 430070, China;

[4] Laboratory of Environment and Livestock Products, Henan University of Science and Technology, Luoyang 471023, China.

[*] **Corresponding author:**

Dr. Fang Liu
No.15 Longzihu University Area, Zhengdong New District, Henan Agricultural University, Zhengzhou 450046, China.
E-mail: liufang.vet@henau.edu.cn
Tel: +86-13903831328

Supplemental materials

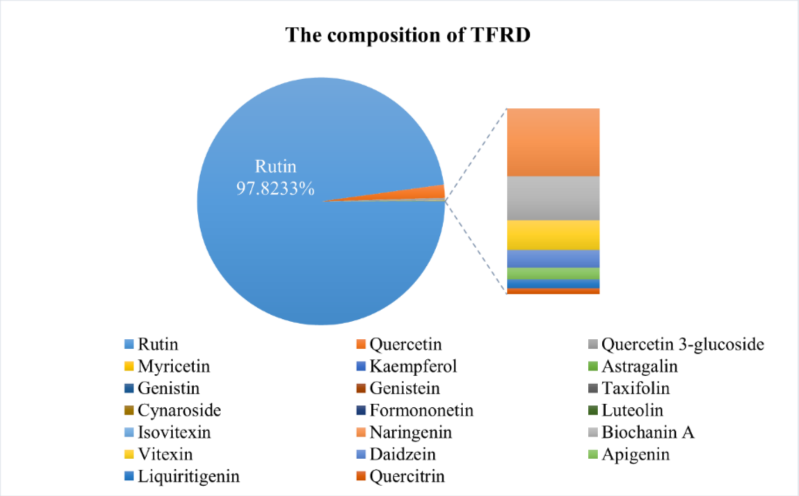

Fig. S1 Composition and percentage of components of TFRD.

Table S1 Composition and percentage of components of TFRD.

Composition	Percentage (%)
Rutin	97.8233
Quercetin	1.7228
Quercetin 3-glucoside	0.2727
Myricetin	0.0468
Kaempferol	0.0365
Astragalin	0.0362
Genistin	0.0193
Genistein	0.0116
Taxifolin	0.0069
Cynaroside	0.0054
Formononetin	0.0045
Luteolin	0.0044
Isovitexin	0.0032
Naringenin	0.0023
Biochanin A	0.0015
Vitexin	0.001
Daidzein	0.0006
Apigenin	0.0004
Liquiritigenin	0.0003
Quercitrin	0.0002

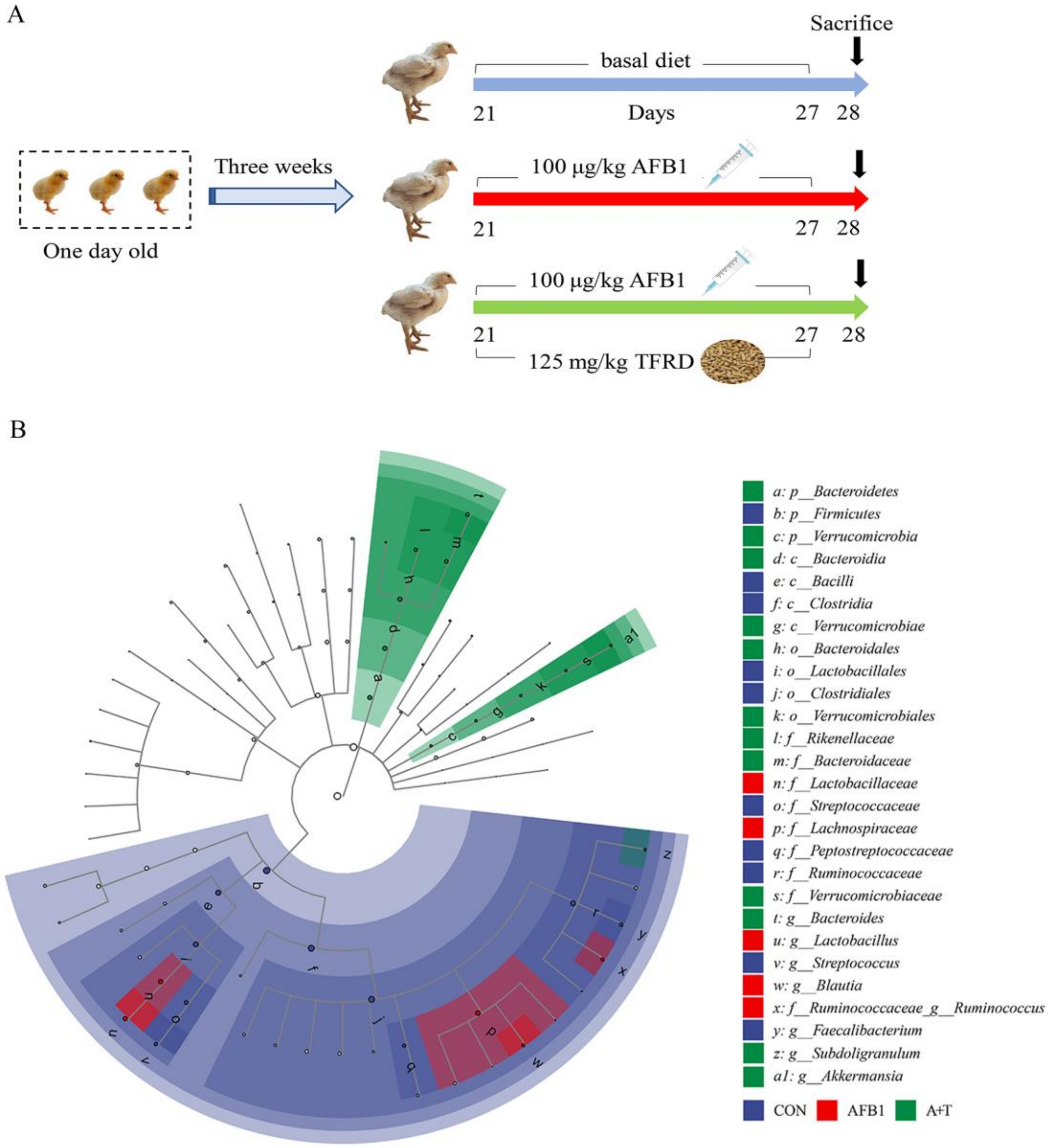

Fig. S2 (**A**) Scheme of experiments. (**B**) Cladogram generated from LEfSe analysis of gut microbiota (LDA score > 3.5).

Table S2 Primer sequences for quantitative real-time PCR analysis.

Gens	Gene bank ID	Primer sequence (5'-3')	Products length
ACSL1	XM_046916038.1	F: GACTAATGGTCACAGGAGCAGCAC	133
		R: CCAGGCATTGACAGTGAGCATCC	
ACSL4	XM_046917350.1	F: CAATAGAGCAGAGTACCCTGAG	146
		R: TAGAACCACTGGTGTACATGAC	
ACC	XM_046929960.1	F: TCCAGCAGAACCGCATTGACAC	187
		R: GTATGAGCAGGCAGGACTTGGC	
Claudin-1	NM_001013611.2	F: GGGGACAACATCGTGACCG	100
		R: AGGAGTCGAAGACTTTGCACT	
CPT-1A	XM_046918285.1	F: CACAGGGCTTTGGGTTGC	132
		R: TTTACAAGAGTCATCCACAGCTTGG	
FAS	NM_205155.4	F: GCTCTGCGTCTGCTTCAGTCTAC	96
		R: GGTACAGGACTCTGCCATCAATGC	
FTH1	NM_205086.2	F: GCCGAGAAACTGATGAAGCTGC	113
		R: GCACACTCCATTGCATTCAGCC	
GPX4	NM_204220.3	F: AACCAGTTCGGGAAGCAGGA	181
		R: ACTTGATGGCATTCCCCAGC	
MUC2	XM_040673077.2	F: CAGGATACGTGTGTGCCCAT	198
		R: GGACGCGTTGCAATCAAAGT	
Occludin	XM_046904540.1	F: CCTCATCGTCATCCTGCTCT	95
		R: GGTCCCAGTAGATGTTGGCT	
PPARα	XM_046906390.1	F: TGCTGTGGAGATCGTCCTGGTC	166
		R: CTGTGACAAGTTGCCGGAGGTC	
SREBP1	XM_046900546.1	F: TTCTTCGTGGACGGGGATTG	218
		R: AGCTGAAGGTACTCCAACGC	
GAPDH	NM-204305.1	F: CCTCTCTGGCAAAGTCCAAG	176
		R: GGTCACGCTCCTGGAAGATA	

Note: F.Forward primer; R. Reverse primer. ACSL1, acyl-CoA synthetase long chain family member 1; ACSL4, acyl-CoA synthetase long chain family member 4; ACC, acetyl-CoA carboxylase alpha; CPT-1A, carnitine palmitoyltransferase 1A; FAS, fatty acid synthase; FTH1, ferritin heavy chain 1; GPX4, glutathione peroxidase 4; MUC2, mucin2; PPARα, peroxisome proliferator activated receptor alpha; SREBP1, sterol

regulatory element binding transcription factor 1; GAPDH, reference gene.
